# Comparison of state-of-the-art biopsy systems for ultrasound-guided breast biopsy using a chicken breast phantom

**DOI:** 10.1007/s10396-024-01482-4

**Published:** 2024-08-06

**Authors:** Leona Katsuta, Tomoyuki Fujioka, Kazunori Kubota, Mio Mori, Emi Yamaga, Yuka Yashima, Arisa Sato, Mio Adachi, Toshiyuki Ishiba, Goshi Oda, Tsuyoshi Nakagawa, Ukihide Tateishi

**Affiliations:** 1 Department of Diagnostic Radiology, Tokyo Medical and Dental University, 1-5-45 Yushima, Bunkyo-Ku, Tokyo, 113-8510 Japan; 2 Department of Radiology, Dokkyo Medical University Saitama Medical Center, 2-1-50 Minamikoshigaya, Koshigaya, Saitama 343 - 8555 Japan; 3 Department of Radiology, Tokyo Metropolitan Toshima Hospital, 33-1 Sakae-Cho, Itabashi-Ku, Tokyo, 173-0015 Japan; 4 Department of Radiology, Nitobe Memorial Nakano General Hospital, 4-59-16 Chuo, Nakano-Ku, Tokyo, 164-8607 Japan; 5 Department of Surgery, Breast Surgery, Tokyo Medical and Dental University, 1-5-45 Yushima, Bunkyo-Ku, Tokyo, 113-8510 Japan

**Keywords:** Ultrasound, Core needle biopsy, Vacuum-assisted biopsy, Breast cancer, Breast phantom

## Abstract

**Purpose:**

To compare different biopsy systems with different-sized needles by determining the weight of the tissue cores, which is one of the important factors for precise pathological diagnoses, and to provide a rationale for choosing the appropriate breast biopsy system with the appropriate needle for breast cancer biopsy.

**Methods:**

Six different vacuum-assisted biopsy (VAB) systems and one core needle biopsy (CNB) system with different-sized needles in different modes were compared, representing 15 total combinations. Tissue cores were obtained from a chicken breast phantom, which is a common substitute for human breast tissue. Five cores were taken for each combination and weighed.

**Results:**

The CNB combination provided significantly lighter tissue cores compared with the VAB combinations with the same-size (14-G) needle (P < 0.01). The combinations using the thickest needle obtained the heaviest among all systems (P < 0.02). The untethered battery-free VAB system yielded the lightest specimen among the VAB systems with the same-sized (12-G) needle (P < 0.04). The percent coefficient of variation (%CV) of the core weights obtained using VAB without a basket was significantly smaller compared with the core weights obtained using VAB with a basket (P < 0.01).

**Conclusion:**

VAB systems can yield larger tissue cores compared with CNB systems. The size of the tissue cores varies even with the same-sized needle among different VAB systems. When performing a breast tissue biopsy, it is important to consider not only CNB versus VAB but also what specific device to use with which needle size.

## Introduction

Breast cancer is the most common cancer in women. When a suspicious lesion is discovered, it is typically categorized as 4 or 5 according to the Breast Imaging Reporting and Data System (BI-RADS) [1], and an image-guided breast biopsy should be performed. The imaging modalities that are primarily used for the biopsy include ultrasonography (US), mammography (MG), and magnetic resonance imaging (MRI), with the lesion features dictating which is selected. Of these, ultrasound is the most widely used modality for breast biopsy because it enables real-time visualization of lesions, resulting in shorter procedure times and less invasiveness.

A breast biopsy technique is categorized into three types: fine-needle aspiration (FNA), core-needle biopsy (CNB), and vacuum-assisted biopsy (VAB). All of these techniques are commonly used for US-guided breast biopsy. FNA is the easiest and least invasive technique, but provides only a cytological diagnosis; thus, information on the subtype of the lesion is not available. Wang et al. [2] reported that the sensitivity is inferior compared with CNB. If the possibility of malignancy is relatively high based on the lesion features, CNB or VAB is usually performed to confirm or exclude malignancy. In addition, Yashima et al. [3] reported that a pathological assessment for malignancy along with biomarker assays are needed to provide patients with appropriate treatment.

Some companies have developed a variety of biopsy devices with different-sized needles to perform CNB or VAB, particularly in recent models. A thicker needle can yield more tissue, but it costs more [4] and is more invasive. Previous studies [5, 6] indicated how much tissue each system could yield, but there are no descriptions of core weights of tissue obtained with state-of-the-art biopsy systems. Consequently, choosing the appropriate system for a precise pathological diagnosis can be challenging, particularly when considering biopsy techniques and needle sizes.

The purpose of this study was to compare the latest biopsy systems using ultrasound-guided breast biopsy and to select the appropriate breast biopsy system and needle for each clinical case. The results offer patients a more precise diagnosis and treatment with reduced invasiveness and cost. Thus, our findings will contribute to the improved diagnosis and treatment of breast cancer.

## Materials and methods

### Preparation of biopsy devices

Five different VAB systems and one CNB system were used as follows: ATEC^®^ Sapphire™ (Hologic Inc., Bedford, MA, USA), EnCor^®^ Enspire™ (BARD GmbH, Karlsruhe, Germany), EleVation™ (BD, Franklin Lakes, NJ, USA), Vacora™ (BARD GmbH, Karlsruhe, Germany), Celero^®^ (Hologic Inc., Bedford, MA, USA), and Magnum™ (BARD GmbH, Karlsruhe, Germany). These systems are categorized as tethered, in which they are equipped with a console, or untethered. Untethered systems are also categorized as battery-powered or battery-free. Tethered VAB systems include the ATEC^®^ Sapphire™ and EnCor^®^ Enspire™. Untethered battery-powered VAB systems included the EleVation™ and Vacora™. Untethered battery-free systems included the Celero^®^, which is a VAB system, and the Magnum™, which is a CNB system. The systems were equipped with different-sized needles in different modes, if available, with 15 combinations in total: ATEC^®^ Sapphire™ with a 9-gauge (G) or 12-G needle; EnCor^®^ Enspire™ in full-sample or half-sample mode with a 10-G or 12-G needle, respectively; EleVation™ with a 10-G, 12-G, or 14-G needle; Vacora™ with a 10-G or 14-G needle; Celero^®^ with 12-G needle; and Magnum™ with a 14-G, 16-G, or 18-G needle. Vacora™, which is not available on the market, was chosen as a control to compare with the state-of-the-art biopsy systems. Mammotome^®^ is one of the most widely distributed classical breast biopsy systems, but it is not used at our hospital, so we did not include it in this study.

### Preparation of chicken breast

**Fig. 1:**
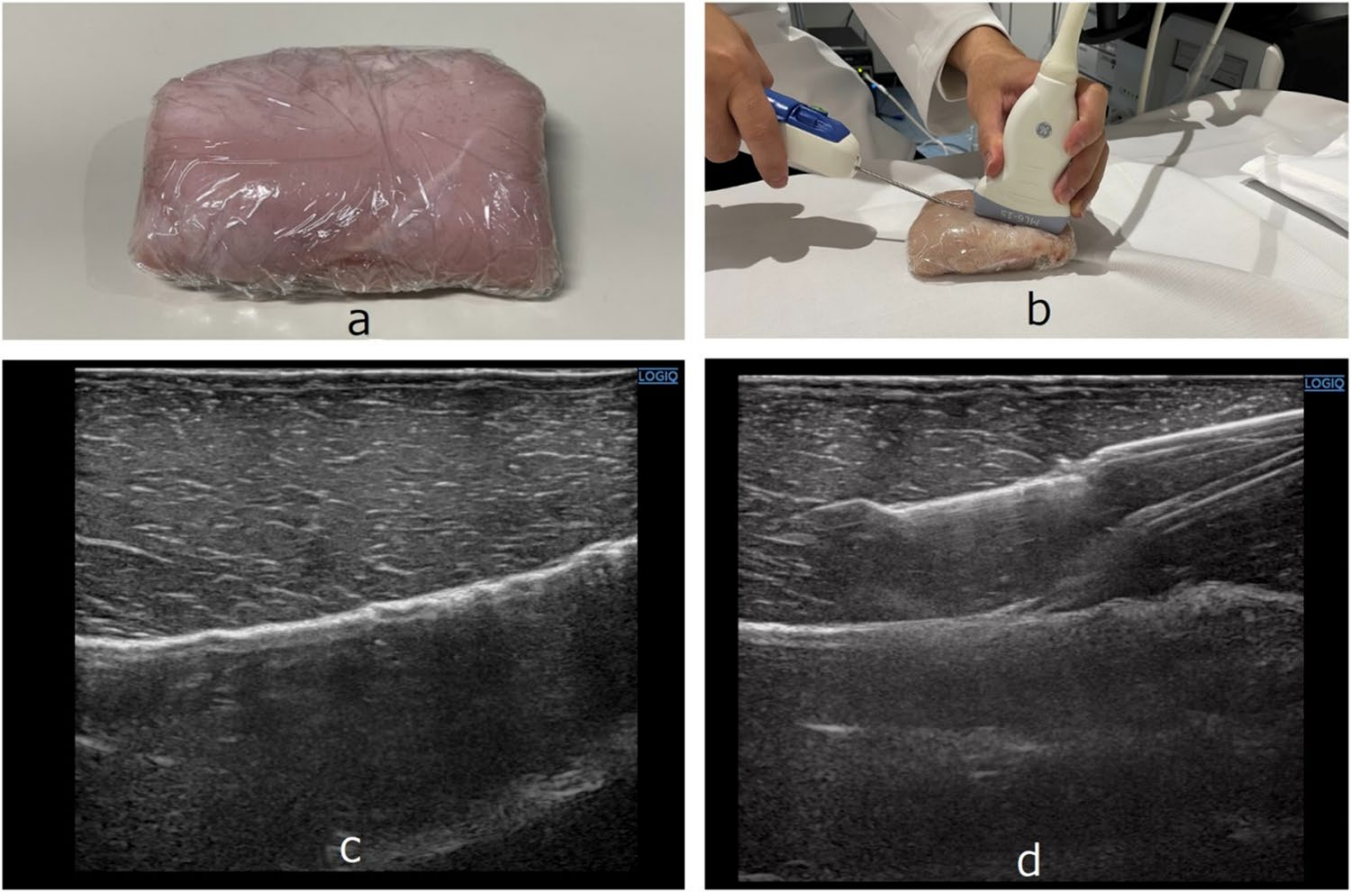
**a** A chunk of chicken breast meat covered with plastic wrap, which was used as a breast phantom; **b** Insertion of a VAB needle into the chicken breast phantom with a US probe; **c** US image of (**a**); **d** US image of (**b**)

We chose chicken breast meat as a human breast phantom. Chicken breast phantoms are commonly substituted for human breast for certain research studies [7–9]. As shown in Fig. 1, we prepared chunks of chicken breast meat covered with plastic wrap as breast phantoms. We selected homogeneous chicken breast meat without fat or air pockets to create a consistent phantom.

### Ultrasound-guided biopsy

A radiologist with 4 years of experience performing CNB and VAB removed five tissue cores from a chicken breast phantom using an ultrasound system (LOGIQ E10s; GE Healthcare, Chicago, IL, USA) and each biopsy system combination shown in Fig. 1. ATEC^®^ Sapphire™, Encor^®^ Enspire™, and EleVation™ were equipped with a basket, enabling us to vacuum several times. The radiologist vacuumed continually five times with only one insertion. Vacora™ and Celero^®^ lack a basket, so they had to be removed from the breast to pick out the tissue core once each vacuum was finished. The radiologist inserted, vacuumed, removed, and picked up the core five times each from separate areas. Five cores were procured from each combination, and the tissue cores were weighed on an ITX120 (AS ONE CORPORATION, Osaka, Japan).

### Statistical analysis

The weights of the tissue cores were measured in milligrams (mg). The mean, standard deviation (SD), and percent coefficient of variation (%CV) of the core weights were calculated for each combination using Microsoft^®^ Excel^®^ for Microsoft^®^ 365 MSO (version 2212 build 16.0.15928.20002) 64-bit (Microsoft, Redmond, WA, USA). Diagrams were created with the same Excel^®^ software. Statistical significance was set at P < 0.05. We assessed only tissue core weights and excluded lengths and volumes because of fragmented cores. We assessed variation without using SD; %CV was used instead because of the dispersion of the mean tissue core weights.

## Results

**Fig. 2:**
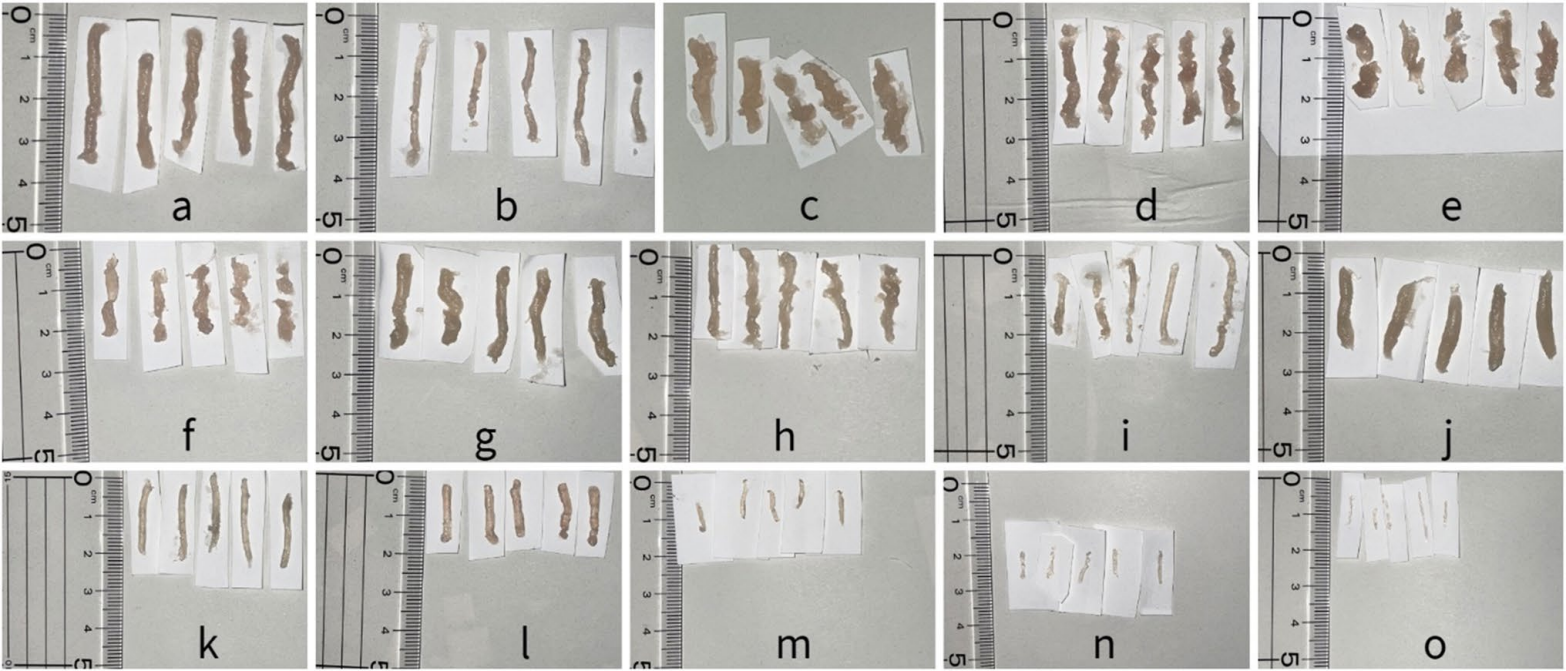
Tissue cores obtained with **a** 9-G ATEC® Sapphire™, **b** 12-G ATEC® Sapphire™, **c** 9-G EnCor® Enspire™ in full-sample mode, **d** 12-G EnCor® Enspire™ in full-sample mode, **e** 9-G EnCor® Enspire™ in half-sample mode, **f** 12-G EnCor® Enspire™ in half-sample mode, **g** 10-G EleVation™, **h** 12-G EleVation™, **i** 14-G EleVation™, **j** 10-G Vacora™, **k** 14-G Vacora™, **l** 12-G Celero®, **m** 14-G Magnum™, **n** 16-G Magnum™, and **o** 18-G Magnum™

**Fig. 3:**
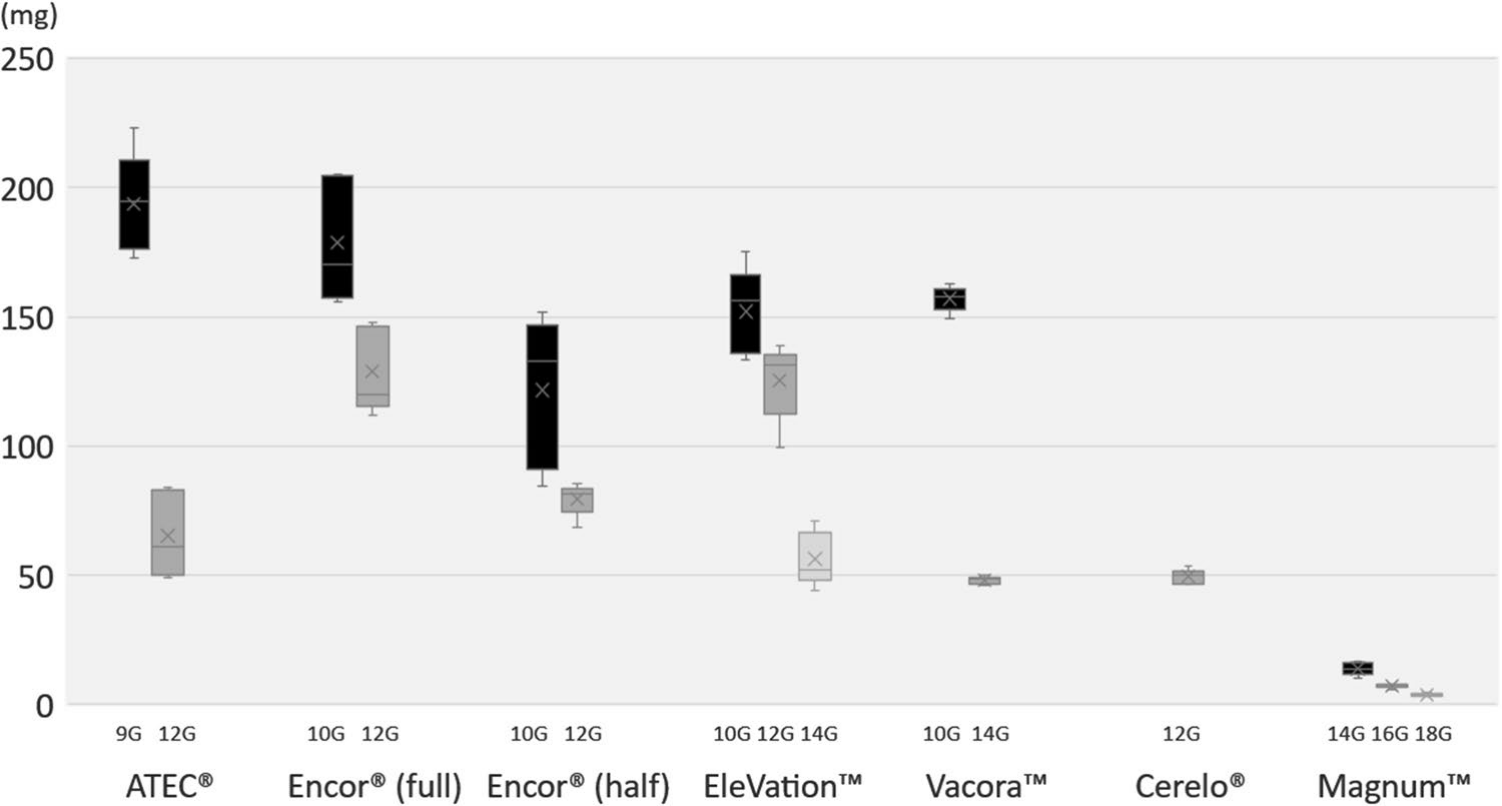
Boxplot of tissue core weights obtained with each biopsy system combination

Figure 2 shows an image of every core obtained from each biopsy system combination. Several cores were partially fragmented. Because this made it difficult to measure the exact length or volume, all tissue cores were weighed. The mean single-core weight ± SD obtained with the 9-G ATEC^®^ Sapphire™ was 193.64 ± 19.30 mg, 65.32 ± 16.73 mg for the 12-G ATEC^®^ Sapphire™, 178.8 ± 24.35 mg for the 9-G EnCor® Enspire™ in full-sample mode, 128.82 ± 16.45 mg for the 12-G EnCor® Enspire™ in full-sample mode, 121.6 ± 29.18 mg for the 9-G EnCor® Enspire™ in half-sample mode, 79.64 ± 8.09 mg for the 12-G EnCor® Enspire™ in half-sample mode, 152.06 ± 16.64 mg for the 10-G EleVation™, 125.42 ± 15.35 mg for the 12-G EleVation™, 56.3 ± 10.49 mg for the 14-G EleVation™, 157.04 ± 4.95 mg for the 10-G Vacora™, 48.06 ± 1.35 mg for the 14-G Vacora™, 49.44 ± 2.76 mg for the 12-G Celero®, 13.9 ± 2.44 mg for the 14-G Magnum™, 7.18 ± 0.81 mg for the 16-G Magnum™, and 3.84 ± 0.62 mg for the 18-G Magnum™. Table 1 lists the mean core weights and %CV for each combination. A boxplot showing the tissue core weights obtained with each biopsy system combination was also prepared (Fig. 3).

**Table 1:** Mean core weights and %CV of each biopsy system combination

Biopsy style	w/ or w/o basket	Biopsy system	Needle size	Mean core weight (mg)	%CV
Tethered (w/ console) VAB	w/ basket	ATEC®	9 G	193.64	9.97
			12 G	65.32	25.60
		Encor® (full)	10 G	178.80	13.60
			12 G	128.82	12.80
		Encor® (half)	10 G	121.60	24.00
			12 G	79.64	8.09
Untethered Battery-powered VAB		EleVation™	10 G	152.06	10.90
			12 G	125.42	12.20
			14 G	56.30	18.60
	w/o basket	Vacora™	10 G	157.04	3.15
			14 G	48.06	2.81
Untethered Battery-free VAB		Celero^®^	12 G	49.44	5.59
Untethered Battery free CNB		Magnum™	14 G	13.90	17.60
			16 G	7.18	11.30
			18 G	3.84	16.10

*VAB* vacuum-assisted biopsy, *CNB* core needle biopsy, *%CV* percent coefficient of variation (%CV)

Comparing same-sized needles, the CNB combination with a 14-G needle (Magnum™) resulted in significantly lighter (P < 0.01) tissue cores than VAB combinations with a 14-G needle (EleVation™, Vacora™), which is listed in Table 2. With respect to combinations on the same system or with the same-sized needle, the thickest needle resulted in the heaviest sample (P < 0.02) among the systems, whereas Celero^®^, which was the only untethered battery-free VAB system in the study, resulted in the lightest (P < 0.04) among the VAB systems with a 12-G needle (Table 3). The %CV of the core weights obtained with VAB systems without a basket were significantly smaller (P < 0.01) compared with those obtained with VAB systems with a basket (Table 4).

**Table 2:** Comparison of VAB and CNB with the same-sized needle (14 G)

Biopsy style	Biopsy system	Mean core weight (mg)
Untethered battery-powered VAB	EleVation™	56.30
	Vacora™	48.06
Untethered battery-free CNB	Magnum™	13.90

VAB, vacuum-assisted biopsy; CNB, core needle biopsy. The CNB combination with a 14-G needle (Magnum™) resulted in significantly lighter tissue cores than VAB combinations with a 14-G needle (EleVation™, Vacora™) (P < 0.01)

**Table 3:** Comparison of VAB with the same-sized needle (12 G)

Biopsy style	Biopsy system	Mean core weight (mg)
Tethered (w/ console) VAB	ATEC^®^	65.32
	Encor^®^ (full)	128.82
	Encor^®^ (half)	79.64
Untethered battery-powered VAB	EleVation™	125.42
Untethered battery-free VAB	Celero^®^	49.44

VAB, vacuum-assisted biopsy. Celero^®^, which was the only untethered battery-free VAB system in the study, resulted in the lightest among the VAB systems with a 12-G needle (P < 0.04)

**Table 4:** Comparison of VAB with or without a basket

Biopsy style	w/ or w/o basket	Biopsy system	Needle size	Percent coefficient of variation
Tethered (w/ console) VAB	w/ basket	ATEC^®^	9 G	9.97
			12 G	25.60
		Encor^®^ (full)	10 G	13.60
			12 G	12.80
		Encor^®^ (half)	10 G	24.00
			12 G	8.09
Untethered Battery-powered VAB		EleVation™	10 G	10.90
			12 G	12.20
			14 G	18.60
	w/o basket	Vacora™	10 G	3.15
			14 G	2.81
Untethered Battery-free VAB		Celero^®^	12 G	5.59

*VAB* vacuum-assisted biopsy, *CNB* core needle biopsy. The percent coefficient of variation of the core weights obtained with VAB without a basket were significantly smaller compared with those obtained with VAB with a basket (P < 0.01)

## Discussion

We obtained tissue cores from a chicken breast phantom using breast biopsy systems used in actual clinical cases. The tissue cores, weights, and fragments depended on needle size, CNB or VAB, and with or without baskets. In recent years, breast imaging diagnostics have advanced with improvements in imaging technology and diagnostic accuracy [10–15]. The effective utilization of artificial intelligence by radiologists is expected to further enhance diagnostic performance [16–19]; however, for selecting treatment, a pathological diagnosis through image-guided biopsy is necessary.

Preibsch et al. [20] reported the mean single turkey breast tissue core weight ± SD obtained with the 8-G original Mammotome^®^, which is one of the most widely distributed classical breast biopsy systems, was 0.192 ± 0.027 g, and 0.084 ± 0.032 g with an 11-G needle. Although we did not include Mammotome^®^ in our study, their results are reasonable considering the needle size used in our research.

Berg et al. [5] reported that 14-G, directional, VAB probes yielded significantly larger specimens compared with 14-G core biopsy guns. Poellinger et al. [6] reported that a vacuum-assisted breast biopsy system with a 9-G needle yielded significantly heavier tissue specimens compared with vacuum-assisted breast biopsy systems with an 11-G needle. Similarly, the results in the present study indicate that VAB systems can yield larger tissue cores compared with CNB systems, even with the same needle size. Each biopsy system with a thicker needle resulted in heavier tissue cores. This result may commonly be accepted by radiologists who have experience in performing CNB and VAB. In terms of cost, Yashima et al. [3] reported a larger volume of specimens can be obtained with VAB compared with CNB, but VAB is more costly.

The results of the present study also indicate that Celero^®^, which is an untethered battery-free VAB system, results in lighter tissue cores compared with VAB systems using a 12-G needle, although they all used the same-sized needle and were all categorized as VAB. As an untethered battery-free VAB system can be used as easily as a CNB system, it is commonly used in many medical facilities for exact pathological diagnoses; however, we considered it inferior to a tethered or untethered battery-powered VAB system in terms of single tissue core weight. Nakano et al. [21] also recommended that US-guided biopsy techniques should be categorized based on the use of tethered or untethered devices.

Tissue cores procured via VAB without a basket were statistically unlikely to vary in terms of core weight. As shown in Fig. 2, the cores procured via the Celero® and Vacora™ without baskets appeared smoother than the ones obtained with other VAB systems. This indicates that tissue cores taken via VAB without a basket are less likely to collapse. Preibsch et al. [20] also reported that the 10-G Vacora™ exhibited the lowest variation in specimens. VAB systems with a basket are superior to those without in terms of invasiveness, because it is not necessary to reinsert the vacuum again; however, with respect to tissue core fragmentation, VAB systems with a basket are inferior to those without. This fact may indicate that friction from the vacuum tube to the basket may fragment cores, although it is hard to prove this hypothesis. A non-mass lesion is a hypoechoic area that has an indistinct shape on two different projections, but that does not fit the criteria of a mass; that is, it lacks convex outer borders and conspicuity [22–26]. Yashima et al. [3] reported that whether VAB or CNB was chosen for non-mass lesion biopsy affected the appropriate pathological result. In addition to whether CNB or VAB is selected, considering the use of VAB with or without a basket may be one of the important factors for an exact pathological diagnosis, because tissue core fragmentation may complicate an accurate pathological diagnosis, especially for non-mass lesions, which need more tissue for an exact pathological diagnosis than mass lesions.

This study revealed that each breast biopsy system, including brand-new ones with each size needle, may be used to determine how much tissue can be obtained at once with each variation. Thus, the results can guide selection of an appropriate biopsy system. This includes CNB versus VAB with an appropriate needle size and to determine how many passes are necessary to obtain a tissue core. Cho et al. [27] reported that an 11-G VAB device was superior to a 14-G automated gun in terms of underestimation, re-biopsy, and false negative rate. Suh et al. [28] reported that the underestimation rate of invasive carcinoma in cases of ductal carcinoma in situ for US-guided core biopsies was significantly higher for CNB versus VAB. These results may also lead to a reduction in excessively frequent biopsies using inappropriately thin needles. Furthermore, shorter biopsy procedures, specifically those that can be completed in less time, can provide patients with a more comfortable experience and fewer complications. However, when deciding on a biopsy method, it is essential to consider several factors, including diagnostic certainty, the costs required for the biopsy, and the patient's background (such as bleeding tendencies, use of anticoagulants or antiplatelet medications, financial situation, and overall physical condition to withstand an invasive procedure. Solid benign tumors like fibroadenomas or solid malignant tumors with minimal necrosis and hemorrhage can often be accurately diagnosed with CNB based on our experience. However, there are no comprehensive studies or guidelines that clearly indicate which cases are more appropriate for CNB over VAB, highlighting the need for future investigations to determine the best approach for specific cases.

One of the limitations to this study was that this research was conducted at only one institution. It is surely preferable to conduct such a study at multiple institutions to reduce various types of bias or factors. However, there are significant logistical and financial constraints that make this approach challenging. Another limitation to this study was that we used chicken breast as a phantom, the uniformity of which has not been standardized in previous research. Thus, we should consider whether the results are applicable to actual clinical cases. In the future, we will need to use human breast tissue as a breast phantom to evaluate the various biopsy systems.

## Conclusion

VAB systems can yield a larger tissue core compared with CNB systems. The size of a tissue core obtained with each VAB system varies, even with the same-sized needle. VAB systems with a basket are preferable because it is not necessary to reinsert the vacuum several times, which is less invasive; however, the resulting cores are likely to become fragmented. When a breast tissue biopsy is performed, it is important to consider not only CNB versus VAB but also to determine which needle size to use.

## Data Availability

Data of this review can be made available upon request.

## References

[1] D'Orsi CJ, Sickles EA, Mendelson EB, et al. ACR BI-RADS Atlas, Breast Imaging Reporting and Data System, 5th ed. Reston, VA: American College of Radiology. 2013

[2] Wang M, He X, Chang Y, et al. A sensitivity and specificity comparison of fine needle aspiration cytology and core needle biopsy in evaluation of suspicious breast lesions: a systematic review and meta-analysis. Breast. 2017;31:157–66. 27866091 10.1016/j.breast.2016.11.009

[3] Yashima Y, Fujioka T, Kubota K, et al. Comparison of the clinical and pathological characteristics of ultrasound-guided biopsy for breast masses and non-mass lesions between 16-gauge spring-loaded core needle biopsy and 12-gauge spring-loaded vacuum-assisted biopsy. J Med Ultrason. 2023;50:205–12. 10.1007/s10396-022-01279-3 PMC11976328 36645627

[4] Grady I, Vasquez T, Tawfik S, et al. Ultrasound-guided core-needle versus vacuum-assisted breast biopsy: a cost analysis based on the American society of breast surgeons’ mastery of breast surgery registry. Ann Surg Oncol. 2017;24:676–82. 27714540 10.1245/s10434-016-5607-3

[5] Berg WA, Krebs TL, Campassi C, et al. Evaluation of 14- and 11-gauge directional, vacuum-assisted biopsy probes and 14-gauge biopsy guns in a breast parenchymal model. Radiology. 1997;205:203–8. 9314986 10.1148/radiology.205.1.9314986

[6] Poellinger A, Bick U, Freund T, et al. Evaluation of 11-gauge and 9-gauge vacuum-assisted breast biopsy systems in a breast parenchymal model. Acad Radiol. 2007;14:677–84. 17502257 10.1016/j.acra.2007.02.013

[7] Choridah L, Kurniadi D, Ain K, et al. Comparison of electrical impedance tomography and ultrasonography for determination of solid and cystic lesion resembling breast tumor embedded in chicken phantom. J Electr Bioimpedance. 2021;12:63–8. 34966468 10.2478/joeb-2021-0008 PMC8667814

[8] Seow JH, Phillips M, Taylor D. Sonographic visibility of breast tissue markers: a tissue phantom comparison study. Australas J Ultrasound Med. 2012;15:149–57. 28191161 10.1002/j.2205-0140.2012.tb00198.x PMC5024915

[9] Zhao X, Ersoy E, Ng DL. Comparison of low-cost phantoms for ultrasound-guided fine-needle aspiration biopsy training. J Am Soc Cytopathol. 2023;12:275–83. 37085429 10.1016/j.jasc.2023.03.005 PMC10330098

[10] Yamaguchi K, Nakazono T, Egashira R, et al. Maximum slope of ultrafast dynamic contrast-enhanced MRI of the breast: comparisons with prognostic factors of breast cancer. Jpn J Radiol. 2021;39:246–53. 33001328 10.1007/s11604-020-01049-6

[11] Honda M, Kataoka M, Kawaguchi K, et al. Subcategory classifications of breast imaging and data system (BI-RADS) category 4 lesions on MRI. Jpn J Radiol. 2021;39:56–65. 32870440 10.1007/s11604-020-01029-w

[12] Li X, Chai W, Sun K, et al. The value of whole-tumor histogram and texture analysis based on apparent diffusion coefficient (ADC) maps for the discrimination of breast fibroepithelial lesions: corresponds to clinical management decisions. Jpn J Radiol. 2022;40:1263–71. 35793052 10.1007/s11604-022-01304-y

[13] Nara M, Fujioka T, et al. Prediction of breast cancer risk by automated volumetric breast density measurement. Jpn J Radiol. 2023;41:54–62. 35913644 10.1007/s11604-022-01320-y

[14] Satoh Y, Imai M, Ikegawa C, Onishi H, et al. Image quality evaluation of real low-dose breast PET. Jpn J Radiol. 2022;40(11):1186–93. 35612727 10.1007/s11604-022-01293-y PMC9616787

[15] Terada K, Kawashima H, Yoneda N, et al. Predicting axillary lymph node metastasis in breast cancer using the similarity of quantitative dual-energy CT parameters between the primary lesion and axillary lymph node. Jpn J Radiol. 2022;40:1272–81. 35877033 10.1007/s11604-022-01316-8 PMC9719886

[16] Ozaki J, Fujioka T, Yamaga E, et al. Deep learning method with a convolutional neural network for image classification of normal and metastatic axillary lymph nodes on breast ultrasonography. Jpn J Radiol. 2022;40:814–22. 35284996 10.1007/s11604-022-01261-6

[17] Uematsu T, Nakashima K, Harada TL, et al. Comparisons between artificial intelligence computer-aided detection synthesized mammograms and digital mammograms when used alone and in combination with tomosynthesis images in a virtual screening setting. Jpn J Radiol. 2023;41:63–70. 36068450 10.1007/s11604-022-01327-5 PMC9813079

[18] Ueda D, Yamamoto A, Takashima T, et al. Visualizing “featureless” regions on mammograms classified as invasive ductal carcinomas by a deep learning algorithm: the promise of AI support in radiology. Jpn J Radiol. 2021;39:333–40. 33200356 10.1007/s11604-020-01070-9

[19] Ishihara M, Shiiba M, Maruno H, et al. Detection of intracranial aneurysms using deep learning-based CAD system: usefulness of the scores of CNN’s final layer for distinguishing between aneurysm and infundibular dilatation. Jpn J Radiol. 2023;41(2):131–41. 36173510 10.1007/s11604-022-01341-7 PMC9889446

[20] Preibsch H, Baur A, Wietek BM, et al. Vacuum-assisted breast biopsy with 7-gauge, 8-gauge, 9-gauge, 10-gauge, and 11-gauge needles: how many specimens are necessary? Acta Radiol. 2015;56:1078–84. 25232187 10.1177/0284185114549224

[21] Nakano S, Imawari Y, Mibu A, et al. Differentiating vacuum-assisted breast biopsy from core needle biopsy: is it necessary? Br J Radiol. 2018;91:20180250. 29975150 10.1259/bjr.20180250 PMC6319844

[22] Uematsu T. Non-mass lesions on breast ultrasound: why does not the ACR BI-RADS breast ultrasound lexicon add the terminology? J Med Ultrason. 2023;50:341–6. 10.1007/s10396-023-01291-1 PMC10354162 36905493

[23] Ito T, Ueno E, Endo T, et al. The Japan society of ultrasonics in medicine guidelines on non-mass abnormalities of the breast. J Med Ultrason. 2023;50:331–9. 10.1007/s10396-023-01308-9 PMC10354171 37261555

[24] Kubota K, Mori M, Fujioka T, et al. Magnetic resonance imaging diagnosis of non-mass enhancement of the breast. J Med Ultrason. 2023;50:361–6. 10.1007/s10396-023-01290-2 PMC10353960 36801992

[25] Goto M, Sakai K, Toyama Y, et al. Use of a deep learning algorithm for non-mass enhancement on breast MRI: comparison with radiologists’ interpretations at various levels. Jpn J Radiol. 2023;41:1094–103. 37071250 10.1007/s11604-023-01435-w PMC10543141

[26] Kubota K, Fujioka T, Tateishi U, et al. Investigation of imaging features in contrast-enhanced magnetic resonance imaging of benign and malignant breast lesions. Jpn J Radiol. 2024. 10.1007/s11604-024-01551-1. 38503998 10.1007/s11604-024-01551-1 PMC11217097

[27] Cho N, Moon WK, Cha JH, et al. Sonographically guided core biopsy of the breast: comparison of 14-gauge automated gun and 11-gauge directional vacuum-assisted biopsy methods. Korean J Radiol. 2005;6:102–9. 15968149 10.3348/kjr.2005.6.2.102 PMC2686400

[28] Suh YJ, Kim MJ, Kim EK, et al. Comparison of the underestimation rate in cases with ductal carcinoma in situ at ultrasound-guided core biopsy: 14-gauge automated core-needle biopsy vs 8- or 11-gauge vacuum-assisted biopsy. Br J Radiol. 2012;85:e349–56. 22422382 10.1259/bjr/30974918 PMC3587071

